# Comparison of Patient's Satisfaction Level after Different Types of Posterior Chamber Intraocular Lens Implantation

**DOI:** 10.5402/2012/629158

**Published:** 2012-06-19

**Authors:** Muhammad Waseem, Sadia Humayun, Omer Farooq, Quratulain Humayun, Sana Sadiq Sheikh

**Affiliations:** ^1^Eye Department, PNS SHIFA Karachi, Karachi 75530, Pakistan; ^2^PNS SHIFA Karachi, Karachi 75530, Pakistan; ^3^AKUH Karachi, Karachi 3500, Pakistan

## Abstract

*Objective*. To compare patient's satisfaction level in performing routine activities during daylight and night vision after implantation with rigid, foldable, or rollable posterior chamber intraocular lens implants in uneventful cataract surgery. *Design*. Retrospective, cross-sectional. Place and Duration of Study. PNS SHIFA Hospital, Karachi, from Nov. 2009 to Nov. 2010. *Methodology*. 91 cataract surgery patients who had uneventful phacoemulsification, within the bag placement of intraocular lens and achieved best corrected visual acuity 6/9 or better were included in the study. Patients who developed postoperative complications were excluded. A specially designed questionnaire was used to assess patient's satisfaction level of vision for those who underwent cataract surgery at least 3 months ago. Finally, they were categorized into five groups ranging from “very good” to “very poor.” SPSS version 16 was used to analyze the results. Results. There was a difference in satisfaction level between three groups. Vision was good in the day and the night with foldable posterior chamber intraocular lens implants. *Conclusion*. It was concluded that visual satisfaction level of patients who had foldable posterior chamber intraocular lens implantation was better during the day and night as compared to patients who had rigid or rollable posterior chamber intraocular lenses implantation.

## 1. Introduction

Cataract is the most important cause of reversible visual impairment [1]. In Pakistan approximately 570,000 adults are estimated to be blind as a result of cataract [2]. Surgical removal of the cloudy lens and implantation of an artificial synthetic ocular lens is usual treatment of a cataract. With advancement in technology, now flexible lenses either rollable or foldable are available. Insertion of these lenses requires a smaller incision than a rigid lens, thus providing for a more gentle surgical intervention [3]. Rigid lenses are now largely being replaced by flexible lenses. In Germany the percentage of rigid lenses implanted was under 1% in 2007 [4]. 

Studies have assessed the success of any type of surgery by rate of complications in the course of the procedure, visual acuity, and long-term visual result. Most common outcomes of interest are high-contrast visual acuity and the residual refraction deficit at the visual distances for which the surgery was intended. But it has long been recognized that the quality of vision before and after surgery is a better measure of the surgical result than visual acuity alone. Quality of vision is the patient's ability to see well in the context of his or her own individual visual requirements, that is, patient's satisfaction with the surgery. In this study we aim to assess patient's satisfaction levels of vision having different type of posterior chamber intraocular lens implants using rigid, foldable, and rollable intraocular lenses, in both day and night. 

## 2. Methodology

This retrospective, cross-sectional study was conducted at Eye department, PNS SHIFA Karachi, for one year, from Nov. 2009, to Nov., 2010 and quota sampling was done. Approval from hospital ethical committee was obtained. Informed written consent was taken. Patients who underwent cataract surgery at least 3 months ago were included in the study. Type of IOL implantation was noted from the documents. Patients who had complications during surgery were excluded from the study. Best corrected visual acuity (BCVA) was recorded. Patients with BCVA less than 6/9 or N8 in the operated eye were excluded from the study. Complete ophthalmic examination including anterior and posterior segment and IOP measurement was done. Pupils were dilated with tropicamide 1% to see in the bag placement of IOL. Postoperative complications that can affect quality of vision including PCO and CMO were excluded from the study. A specially designed questionnaire was used to assess the patient satisfaction level of vision. Glare was assessed by trouble seeing street signs during daylight and problems with oncoming headlights at night. Colour perception was assessed by asking for trouble recognizing specific colours. Depth perception was assessed by trouble pouring liquids or going downstairs. Haloes were assessed by asking for rings around light during night. Quality of vision was assessed during TV watching, playing or working outdoor, reading time on watch and wall clock, driving at night and during rain, and using computer and cell phone. After asking all the above questions the patients were asked to give an overall satisfaction level. Satisfaction level was categorized into “very good,” “good,” “satisfactory,” “poor” and “very poor.” Type of posterior chamber lens implant (rollable, foldable, or rigid) was the independent variable. Age, sex of the patient, education level, and complications of the surgery were the possible covariates. 

Data was entered twice in the statistical software (EpiData and SPSS version 16.0) and errors were checked and corrected. Frequencies and proportions were computed to present all categorical variables. Means and medians were computed for continuous variables.

Mann-Whitney *U* test was applied to compare the proportions of nominal variables (e.g., sex) and ordinal variables (satisfaction level of patients) at *P* < 0.05 level of significances. Comparison of categorical variables (e.g., types of lens) and ordinal variables (satisfaction level of patients) was done using Kruskal Wallis nonparametric test, at 5% significance level. Bonferroni-Holm correction was done as a post hoc test.

Potential sources of bias such as selection bias, interviewer bias, and reporting bias was catered for as much as possible by selecting the participants carefully and proper training of the research assistant for eligibility screening, taking consent and administering the interview. The data collection was done under the supervision of the principle investigator. The data was checked and edited by the investigators.

## 3. Results

125 cataract surgery follow-up patients were assessed for eligibility in 2-month duration of the study. 20 patients did not meet the eligibility criteria and 14 refused to participate in the study. 91 cataract surgery patients agreed to participate and were enrolled in the study. The ocular examination of the participants was done, visual acuity assessed, and questionnaire administered.

The descriptive characteristics of the patients in the three groups are given in Table 1. The three groups were not comparable with respect to education level and sex.

Kruskal-Wallis and Mann-Whitney *U* test were done to assess whether the three types of lens groups are different from each other or not.  Table 2 shows the results of the tests done.

The satisfaction levels during day and night were significantly different for the three lens types (*P* value 0.021 and 0.019). On further analysis by Bonferroni-holm correction, foldable lens was found to be significantly different from the rigid and rollable lens in terms of reported satisfaction level during day and night. The patients who had posterior chamber foldable lens had better visual acuity and satisfaction levels during both day and night as compared to patients with rigid and roll-able lens implants.

The univariate analysis for comparison of different education levels among patients and their reported satisfaction level during day and night did not show significant results (*P*-value 0.636 and 0.422, resp.). Similarly the satisfaction level during day and night was not different for males and females (*P*-value 0.274 and 0.353, resp.), and for right or left eye (*P*-value 0.16 and 0.355, resp.), as assessed through mann-Whitney *U* test.

## 4. Discussion

In this study we aimed to assess patients' satisfaction level with implantation of rigid, foldable, and roll-able lenses. Though rigid lenses have largely been replaced by foldable and roll-able lenses, foldable and roll-able lenses are costly compared to the rigid lenses. For any ailment, treatment advised should not only be effective but it is important to choose most cost-effective available treatment. In our study there was significant difference in patient's vision (*P*-value 0.020 and 0.019 for day vision and night, resp.) with different type of lenses used in cataract surgery (Table 2). Patients who had foldable lens implants were more satisfied compared to patients who had rigid or roll-able lens implants. This finding is incoherent with other studies. Parihar et al. compared the complication rates and visual acuity in patients with roll-able and foldable lens implants. They found that visual acuity was better in patients with roll-able lens implant compared to foldable lens [5]. Reason given to support this finding of better results with roll-able lenses is that insertion of roll-able lenses requires small incision (0.9 millimeter) as compared to foldable lenses (3.0 mm). Small incision means less chances of leak postoperatively and fewer infections [6]. Our data showed that in both males and females there was no difference in vision satisfaction in day (*P*-value 0.544) or night (0.700). But study done by Lundqvist found that gender significantly affects patient's satisfaction. According to their findings women assess their vision worse than males [7]. Our results are contradictory to Lundqvist's findings. Also no significant difference was found in satisfaction level in day or night vision with different levels of education. This result is coherent with study done by Chang-Godinich et al. who also did not find difference in vision and patient satisfaction after surgery with respect to sociodemographic characteristics [8]. Also difference in day or night vision satisfaction was insignificant for being operated on left, right or both eyes. 

Strength of this study is that it has taken wide age range of patients and also both congenital and acquired cataract. Limitation of study is that we cannot tease out the effect of type of lens on patient's vision's satisfaction according to type of cataract, that is, congenital or acquired cataract. Another limitation is that we had not collected data on preexisting eye disease before cataract surgery; this has shown to affect the perceived vision after surgery [9].

## Figures and Tables

**Table 1:** Descriptive statistics (demographic characteristics of three groups).

Characteristic	Over all	Type of posterior chamber lens implant		
		Rigid (*n* = 38) *n* (%)	Foldable (*n* = 26) *n* (%)	Roll able (*n* = 27) *n* (%)
Sex				
Male	50 (54.9)	24 (63.2)	16 (61.5)	10 (37)
Female	41 (45.1)	14 (45.1)	10 (38.5)	17 (63)
Education level				
Under matric	27 (29.7)	16 (42.1)	8 (30.8)	3 (11.1)
Matric/inter-matric	31 (34.1)	16 (42.1)	7 (26.9)	8 (29.6)
Bachelor	19 (20.9)	4 (10.5)	6 (23.1)	9 (33.3)
Masters	14 (15.4)	2 (5.3)	5 (19.2)	7 (25.9)
Operated eye sides				
Right eye	37 (40.7)	25 (65.8)	14 (53.8)	13 (48.1)
Left eye	24 (26.4)	13 (34.2)	12 (46.2)	14 (51.9)

**Table 2:** Satisfaction level during day and night with respect to different lens types.

Characteristics	Over all	Type of posterior chamber lens implant			Significance (*P* value)
		Rigid (*n* = 38) *n* (%)	Foldable (*n* = 26) *n* (%)	Roll able (*n* = 27) *n* (%)
Satisfaction level (day)					**0.02**
Very good	22 (24.2)	6 (15.8)	10 (38.5)	2 (22.2)	
Good	44 (48.4)	19 (50)	14 (53.8)	11 (40.7)	
Satisfactory	20 (22)	11 (28.9)	1 (3.8)	8 (29.6)	
Poor	5 (5.5)	2 (5.3)	1 (3.8)	2 (7.4)	
Satisfaction level (night)					**0.019**
Very good	15 (16.5)	4 (10.5)	7 (26.9)	4 (14.8)	
Good	41 (45.1)	15 (39.5)	14 (53.8)	12 (44.4)	
Satisfactory	27 (29.7)	14 (36.8)	5 (19.2)	8 (29.6)	
Poor	8 (8.8)	5 (13.2)	0	3 (11.1)	
